# From hospital-centered care to home-centered care of older people: propositions for research and development

**DOI:** 10.1108/JHOM-03-2023-0077

**Published:** 2024-01-24

**Authors:** Bonnie Poksinska, Malin Wiger

**Affiliations:** Department of Management and Engineering, Linkopings Universitet, Linkoping, Sweden; Production Development Unit, Region Ostergotland, Linkoping, Sweden

**Keywords:** Care of older people, Person-centered care, Home care, Integrated care, Co-creation, Modularity, Proactive care

## Abstract

**Purpose:**

Providing high-quality and cost-efficient care of older people is an important development priority for many health and social care systems in the world. This paper suggests a shift from acute, episodic and reactive hospital-centered care toward longitudinal, person-centered and proactive home-centered care. The purpose of this paper is to contribute to the knowledge of a comprehensive development strategy for designing and providing home-centered care of older people.

**Design/methodology/approach:**

The study design is based on qualitative research with an inductive approach. The authors study development initiatives at the national, regional and local levels of the Swedish health and social care system. The data collection methods included interviews (*n* = 54), meeting observations (*n* = 25) and document studies (*n* = 59).

**Findings:**

The authors describe findings related to policy actions and system changes, attempts to achieve collaboration, integration and coordination, new forms of care offerings, characteristics of work settings at home and differences in patients' roles and participation at home and in the hospital.

**Practical implications:**

The authors suggest home-centered care as a solution for providing person-centered and integrated care of older people and give examples of how this can be achieved.

**Originality/value:**

The authors outline five propositions for research and development related to national policies, service modularity as a solution for customized and coordinated care, developing human resources and infrastructure for home settings, expanding services that enable older people living at home and patient co-creation.

## Introduction

Health care provision changes with time, and in the past, the primary focus has been on treating acute and episodic illnesses (Lillrank, 2018). Patients experienced health problems only temporarily and care provisions were limited in both time and scope (RAND, 2012). Today, healthcare systems are increasingly tasked with caring for patients with chronic conditions, where the objectives extend to lifelong wellbeing and prevention of complications, rather than solely curing episodic illnesses (Rijken *et al*., 2017). This shift is particularly crucial in the care of older people, as many chronic conditions are closely linked to aging (Briggs *et al*., 2018). In line with this change, healthcare structures, processes and working methods must be renewed and developed. The emphasis is shifting from treating episodic illness in hospitals to developing robust care structures and services for chronic disease management in outpatient settings (WHO, 2016).

Current research and development efforts in healthcare are predominantly aimed at addressing challenges such as increased hospital costs, long waiting times and poor (Brown, 2018). These development initiatives can be described as hospital-centered, as they focus on streamlining patient flows, coordinating care processes, planning for care transitions and discharges, with the aim of reducing emergency department crowding, hospitalizations, readmissions and length of stay (Bazemore *et al*., 2018).

Integrated care models represent a distinct research stream that focuses on creating connectivity, alignment and collaboration between primary, secondary and community care. The goal is to enhance quality of care and quality of life for individuals with chronic conditions (WHO, 2016; Goodwin *et al*., 2017; Damery *et al*., 2016). Previous research focuses on integration of care in community, primary and hospital settings (WHO, 2016; Béland and Hollander, 2011; Machta *et al*., 2019). Our research extends this focus to include the integration of home as a care setting. Since homes are not traditional care institutions, new organizational and managerial mechanisms are needed to ensure coordination and high-quality home-centered care (Rijken *et al*., 2017).

This paper advocates for a shift from acute, episodic and reactive hospital-centered care to a more longitudinal, person-centered and proactive home-centered approach. We define home-centered care as a strategy that designs and delivers services based on a comprehensive understanding of an individual’s needs, values and preferences, allowing older people to remain at home, with hospital care included as necessary. Based on empirical studies of development initiatives in Sweden and integrating theories from logistics, quality, service and healthcare management, we identify five development areas that are important in leveraging more fundamental, systemic and structural changes in providing care of older people. Hence*, the purpose of this paper is to contribute to the knowledge of a comprehensive development strategy for designing and providing home-centered care of older people*.

## Conceptual framework

The foundation of this paper is the ongoing transformation of the Swedish healthcare system towards person-centered and close care (SOU, 2019). Various reforms and policies have been introduced to facilitate this transition. The Swedish Association of Local Authorities and Regions describes the transformation with four goals (SALAR, 2022):

1. from focus on organization to focus on person and relationship
2. from residents and patients as passive recipients to active co-creators
3. from isolated care interventions to coordination based on the person's needs
4. from reactive to proactive care and health promoting

In the following sections we describe research paradigms and recent knowledge developments that form a conceptual framework of this paper and are important for achieving Sweden’s transformation towards a person-centered and close care.

### Focus on person and patients as active co-creators

The first two goals of the transformation emphasize person-centeredness, a concept that has garnered significant attention in recent healthcare management literature (Rosengren *et al*., 2021). Traditionally, Swedish patients have both a passive and a subordinate role and are all too often not sufficiently involved in their own care (Elg *et al*., 2012). In contrast, the person-centered approaches recognize patients as active participants in their care underlining the significance of patient involvement in healthcare improvement (Rosengren *et al*., 2021; McColl-Kennedy *et al*., 2017). In service management literature, customers are seen as active participants in service design and delivery, co-creating value alongside service providers and other actors (Grönroos and Voima, 2013). This co-creation of services involves collaborative activities in the customer-provider sphere, potentially leading to both beneficial and counterproductive outcomes through resource integration on both the provider and customer sides (Oertzen *et al*., 2018). Further, Swedish research indicates that older people want to be actively involved in decisions regarding their care (Jobe *et al*., 2019). However, in hospital-centered care the patient is often subject to production, or at most the co-producer of value but not a co-creator (Elg *et al*., 2012). In home-centered care, the care providers are not producers of value but only facilitators of the value creation process. Hence, caregivers co-create value with patients and their relatives, but patients can also create value themselves (Grönroos and Voima, 2013; McColl-Kennedy *et al*., 2017). The co-creation of healthcare is a central concept for this paper as it promotes the shift from hospital-centered care, largely built around institutions and health professionals, to home-centered care that is designed around a person. While much literature has focused on the need for more person-centered care, there is limited research on how to effectively implement it. This paper aims to contribute to this gap in knowledge.

### Customization and coordination based on the person's needs

Care for older persons often takes place across several care settings involving primary, secondary and community care (Marino *et al*., 2018). In hospital-centered care, healthcare professionals frequently operate in “silos” and provide isolated care interventions without the necessary integration among various care providers (Rijken *et al*., 2017; Poksinska, 2018). This often leads to poor transitions between healthcare providers, coordination deficiencies and a lack of continuity in care (Stiernstedt, 2016; He and Tang, 2021). Each caregiver operates within their own sphere of responsibility, but no single entity is accountable for the entirety of a patient's care (Berglund *et al*., 2015). The need for care coordination is even more pronounced in home-centered care as it encompasses the holistic needs of an older person rather than just an isolated health problem. “Modularity” is an approach that has received a lot of attention in recent years, as it creates favorable conditions for customizing and coordinating care when necessary (Minvielle *et al*., 2014). This approach advocates that systems should be built from cohesive, loosely coupled components, which can be combined in different ways and managed independently (Voss and Hsuan, 2009; Vähätalo and Kallio, 2015; Silander *et al*., 2018). Modularity aims to solve complexity in service systems by enabling efficient customization and responsiveness to individual needs (Voss and Hsuan, 2009). A customized care process is designed for each patient by combining a set of standard components, often provided by different care professionals (De Blok *et al*., 2014). This coordination can be achieved by interfaces, as they deal with interactions between components, how they fit together and how they connect, interact and communicate within modular processes (De Blok *et al*., 2014). These interfaces may include people, information, or rules governing the interplay between modules. Patients and their relatives may also play an important role in the modular design and take responsibility for the arrangement, interconnection and coordination of care services (De Blok *et al*., 2014).

### Proactive care and health promoting

The hospital-centered models in Sweden and Norway suffer from acute healthcare consumption and readmissions because they fail to intervene earlier and identify people at risk before they experience a serious fall requiring hospitalization (Berntsen *et al*., 2019; Marcusson *et al*., 2019). The deficiencies noted, such as poor integration of care, lack of coordination and limited patient access to timely care services, are known risk factors for readmissions (Reed *et al*., 2015). The shift towards home-centered care involves developing proactive approaches that enhance health by mitigating risks and prompting caregivers to take preventive measures (Anell and Glenngård, 2014). Various interventions have been developed and tested in different contexts to address the growing need for preventive measures (WHO, 2018). One example is proactive programs introduced in primary care for frail older patients (Bleijenberg *et al*., 2013; Robinson *et al*., 2021). Primary care centers in Sweden organize personal resources around older people as for example older teams or older nurses to ensure comprehensive and coordinated care (Berglund *et al*., 2015).

Other interventions focus on enabling older individuals to live independently in their homes, a desire shared by many. The number of people receiving health and social care services at home in Sweden has been increasing (SALAR, 2019). Digitalization and modern medical technology have expanded the possibilities for providing high-quality and safe care at home (Lyth *et al*., 2019). Self-monitoring for various chronic conditions like chronic obstructive pulmonary disease (COPD), hypertension, heart failure, or dementia is being introduced and evaluated for older people at home (Chalfont *et al*., 2021). The development of telehealth technologies in Sweden shows promising results for prevention and treatment of chronic diseases, but also challenges related to mistrust in poor information technology (IT) systems, difficulties coping with technology and raising concerns as accessibility to health care (Nymberg *et al*., 2019). Nevertheless, the overall attitude is positive, as older people, empowered with data and information reflecting their personal experiences, become more engaged in managing their health and are more likely to take proactive actions (Gokalp *et al*., 2018).

### Person-centered approaches in international research

Person-centered approaches have long been recognized as critical aspects of caring for older people (Marino *et al*., 2018; Berntsen *et al*., 2019). To meet the increasing healthcare demands of the growing older population, fragmented care systems need to be replaced with person-centered, integrated care models (WHO, 2018). Several change programs have been established, primarily in high-income countries, including Australia, Canada, Italy, the Netherlands, New Zealand, Sweden, the UK and the USA (Hughes *et al*., 2020; Briggs *et al*., 2018). The UK has been a pioneer in this area, with various programs such as case management, older people’s pilots, integrated care pilots, integrated care pioneers and vanguards (Glasby and Miller, 2020). Most improvement initiatives focus on overcoming the shortcomings of traditional hospital-centered care, such as fragmented care and lack of care coordination (Hughes *et al*., 2020). The focus is particularly on micro-level integration of services and organizations, while there is a relative lack of studies on meso-organizational and macro system-level improvement strategies (Briggs *et al*., 2018). Evidence from change programs suggests that any of the improvements pursued in isolation will not be sufficient to make meaningful, measurable changes. Instead, a whole system approach, including all levels of health and social care systems might be needed (Hughes *et al*., 2020). Person-centered approaches are not a unified concept as change programs are rooted in the way that services for older people are financed, governed and organized (Briggs *et al*., 2018). The effectiveness of the person-centered approaches is unclear and questioned as evaluations of benefits and efficiency show mixed results (Marino *et al*., 2018; Rocks *et al*., 2020). However, the differences in the design of change programs and contextual factors are unlikely to affect a predetermined set of outcomes in the same way (Hughes *et al*., 2020).

This paper takes a holistic perspective by studying the development initiatives at national, regional and local level of the Swedish health and social care systems. The concept of home-centered care used in this paper takes a departure from designing care around holistic needs of an older person. The primary focus is not on how to integrate service and organizations of hospital-centered care but how to design and deliver welfare services to older people in a more effective way but not necessarily with reduced costs.

## Method

The study design is based on qualitative research with an inductive approach (Patton, 2002). We collected empirical data on development initiatives related to patient-centered and close care at national, regional and local levels of Swedish health and social care systems.

### Study context

In Sweden, the responsibility for care of older people is divided between regions and municipalities as presented in Table 1. Health and social care are mainly publicly funded with some possibility to contract private providers. Compared to other countries, the whole Swedish healthcare system is highly decentralized. Regions are responsible for providing primary and secondary care and municipalities are responsible for community care. However, the main responsibility for health and social policy lies on the national level.

### Data collection

The paper integrates results from two parallel research projects. Swedish national evaluations showed that policies related to person-centered and close care of older people are not implemented to a sufficient extent (NBHW, 2017). The first project aimed to investigate how national and regional decision makers and management act to stimulate policy uptake at the organizational level. The second research project aimed to develop knowledge about how caregivers involved in the care of older people can be led and organized to promote innovation and new thinking. The main idea was to study local development initiatives, which were distinguished by innovative and more comprehensive structural changes to the way care is provided to older people. The cases were thus at the forefront and unique but aligned with conceivable ones within the landscape of development initiatives in Sweden. The first case study, named “Health city”, was a project co-financed by the European Union (EU) where community, primary and hospital care are integrated and managed through one organization. The second case study, named “Future care”, was a pilot project aiming to develop a new care unit targeting older people and integrate it into the local care system. The last case, “Proactive primary care”, evaluated a new service that identified patients at risk of hospitalization and offered them proactive care services. The regional level was selected based on where the case organizations were located.

Data were collected during the years 2017 to 2021 through interviews (*n* = 54), meeting observations (*n* = 25) and document studies (*n* = 59). An overview of data collection methods, aspects studied and key questions of analysis are presented in Table 2. The interviews were semi-structured and conducted until data saturation was reached. Interviewees were chosen to represent different professions and positions in the case-study organizations. They were nurses, physicians and other care professionals working with older patients in the community, primary and hospital care and managers who are responsible for different caregiving organizations. No patients and informal caregivers were involved in the study. Each interview took about 1–2 h and was taped and transcribed. Participant observations were carried out of meetings related to planning and managing development activities. Meetings were documented using field notes. Studied documents included national and regional strategy documents, activity reports, handling plans, documented procedures and meeting minutes. The documents were retrieved from the webpages of Swedish governmental agencies or provided by the participants of the study.

A case-study protocol including interview guides, transcribed interviews, field notes and a list of documents was established to ensure data reliability (Yin, 2009). Collected data was analyzed using qualitative content analysis (Patton, 2002). The analysis included identifying key concepts as initial coding categories related to key questions of analysis presented in Table 2. The material from interviews, filed notes and documents was very extensive and the focus was on interpreting and summarizing data with the aim of creating nuanced and contextually sensitive results into the research topics. Two researchers coded the data independently and then jointly discussed the results to establish code content consensus.

The study is exempt from ethical approval according to the Swedish legislation on research ethics (SFS (2008:192). Ethical Approval Act of Research Involving Humans. Stockholm). This study was conducted in accordance with the Declaration of Helsinki. Informed consent was obtained from all participants included in the study.

## Results

The study garnered a broad scope of empirical material on development initiatives undertaken at the national, regional and local levels of the Swedish health and social care system. Findings at the national and regional level focused primarily on describing what was undertaken and why. It is a description of the expected outcome rather than an actual outcome of development initiatives. The findings at the local level contain more in-depth insights into the selected aspects (see Table 2) that were considered important for developing home-centered care.

### Policy actions and system changes

Several regulations were enacted over the years to trigger change and enable collaboration between divided healthcare and social care systems. Study documents include several descriptions of policies that were issued to develop person-centered care of older people in Sweden. Three examples of policy changes important for this study are described below.

*Permanent care coordinator* was issued in 2010 (Prop. 2009/10:67) to address patients' needs for security, continuity, coordination and safety in care. The policy gives Swedish patients the right to a contact person that helps them navigate through the system and coordinates care services. Upon request from the patient, the primary care center and hospital clinics appoint a permanent point of contact for the patient.

*Coordinated individual plan (CIP),* also issued in 2010 (Prop. 2009/10:67), aims to ensure patient participation and promote collaboration between healthcare and social care providers. Care providers involved in patient care shall make a holistic assessment of care needs and establish, together with the patient, an individual care plan.

*Integrated discharge from hospital care,* issued in 2018 (Act 2017: 612), aims to ensure a safe and effective transition from inpatient care to a home environment. Integrated discharge means providing support to older patients so they can feel comfortable assuming responsibility for their care and life at home and thus reduces the risk of developing a dependence on care services.

Policies are developed and decided by high level political officials at the national level; however, it is the regional authorities and management of health and social care that are responsible for the local implementation and realization of policies in practice. Region Skåne and their communities signed a special agreement to regulate the responsibilities and liabilities in respect to reaching the goals of policies such as CIP and integrated discharge. Each municipality worked on solutions aimed at realizing the agreement. In the case of “Future care” a collaboration group involving representatives from primary, secondary and community care was established. The people involved included the hospital director, the manager of primary care at the regional level, managers of primary care centers located in the municipality and the head of social care in the community. The primary focus was on removing administrative, financial and structural barriers and establishing processes required for joint service provision. As the three settings differ in terms of their organization, governance, regulation, resources and culture, building a strategic collaboration was considered to be an important mechanism for establishing a common platform to develop care of older people.

In order to succeed it is important to create relationships and trust between our various organizations. It is a huge challenge; in the meanwhile, it is. We all have a mission and I think we have the same ambition. We want this to be as good as possible, but to communicate, to find the ways is not easy … Good cooperation at all levels is a prerequisite. Primary care manager at Region Skåne.

### Collaboration, integration and coordination

The spectrum of caregivers involved in the older people’s care at home in the case studies was immense and involved specialist care, mobile teams, primary care and in-home services. The caregivers belonged to different care settings and had neither the mandate nor the resources necessary to take ownership and responsibility for the entirety of care. Therefore, many collaborative initiatives to establish coordinated and coherent service provision were identified in the study.

In the case of “Future care” the aforementioned strategic collaboration group turned, over time, into to an operative collaboration group involving managers responsible for the provision of different services, for example day care services, discharge teams, or rehabilitation services. The group held monthly meetings and discussed different matters related to the provided services and combining them into a coherent whole. A common issue in development work included discussions about specific person cases with nonconformities and quality issues. This joint discussion contributed to a better understanding of the realities that govern different providers and in identifying the root causes of problems. Solutions involved defining responsibilities, deciding on the course of activities, developing best practices and standardizing communication and information exchange, which is illustrated by the following quote:

It can be, e.g., about how we agree to contact each other. Should we make a phone call, fax or maybe email? It can be such a simple thing. Can we decide on one weekday when we do individual care plans for our patients? It is easier than, when I meet the patient, to say when there is a time next week. I can announce this time to relatives and patients. So, it is very much about who does what and in what order and that we all agreed on this. It is also about telling each other how our organizations work.

The case study “Health city” took a different approach and made an attempt at organizational integration, i.e., gathering and managing all services related to care of older people within a single organizational structure. Despite some initially positive results, complete organizational integration was difficult to achieve. The differences in legal and value systems were a bigger barrier than expected. As result the integration of community services was only symbolic and faced challenges.

The caregivers offered different services that could be customized and combined in different ways, but the challenge was how to navigate and coordinate them with older people. The most common solution was to assign coordinators at different levels with the task of coordinating between caregivers or/and with patients with the overall goal of improving continuity of care. One example is a coordinator that managed handovers and ensured that information was transferred between caregivers. It contributed to informational and management continuity of care, but not relational continuity. The opposite example is a coordinator assigned to a specific person with the task of customizing care services and supporting the older person and his/her relatives during hospital stay and transition to home. Coordinators with this role were considered important to ensure relational continuity of care. A third example is an ‘older pilot’ whose role is helping patients and their relatives to navigate and access different care services. This can also include helping with practical issues such as safety alarms, food deliveries, or personal care.

The required-by-law CIP was used in different services to create customized care plans with an older person and her/his relatives. Although the importance of CIP to ensure effective collaboration and coordination of care was recognized by the caregivers involved, the service was associated with several challenges. The most common challenge was coordinating the time and place to make the plan. There was also a discrepancy between expectations of who should be involved and why. The operative collaboration group often discussed issues related to “not taking responsibility” in creating and realizing plans.

### New forms of care offerings

One of the initiatives studied was *a day care unit* opened in a hospital to offer medical services usually provided in inpatient settings, but which are possible to carry out in outpatient settings. The unit offers services in two main areas: monitoring for clinical decisions and regular day care services. The first service is aimed at patients who arrive at the emergency department, but it is unsure whether they require hospitalization. Patients are systematically monitored to evaluate their medical condition according to disease-specific protocols and to decide about admitting or discharge. The physician working at the unit was employed part-time in the emergency department, which helped to identify patients who could be cared for in this alternative setting. The second area included a wide range of day care services, for example regular intravenous infusions, blood transfusions, draining away excess fluid and monitoring some chronic diseases. These are services that are not provided by primary care and often lead to hospitalization. The day care unit addresses these special needs so older people can remain at home. The day care unit is still in a development stage and the range of services is extended based on arising needs. The patient questionnaire filled out when leaving the unit has consistently shown high satisfaction ratings with the day care services.

Another example is *mobile teams* that offered both acute and planned care services in older persons' home. The mobile team usually consisted of a physician from primary/secondary care and a nurse from community care equipped to perform care services at home. The type of care services varied, but, in many cases, they were perceived as an alternative to hospital admission. A mobile team for planned care could be one of the services decided by the CIP. It was carried out as regular visits with check-ups and reviews of older person’s health. For acute care the mobile team was called when other caregivers visiting the older person regularly considered it to be necessary. Interviewees described mobile teams for planned care as value-adding and an appreciated service by older people. In the case of acute care, opinions were more divided. Inexperienced physicians found that home visits are demanding, as there is limited access to the patient’s medical history, and it is difficult to make appropriate decisions upon meeting a patient only once and not knowing how much the patient's condition has deteriorated. In the case of both mobile teams for acute and planned care, concerns were raised about the economic feasibility and difficulties of integrating services with regular operations in an overloaded primary and secondary care system.

*Discharge teams* were a service developed to realize the law on *integrated discharge from hospital care*. A CIP was drawn up between hospital departments, primary care and community care and the patient on what support should be provided at discharge so the older person can regain their previous functional ability and independence as quickly as possible. It was a time-limited service aiming to convey a sense of security for older people and ensure that their recovery progresses as expected. Depending on the person’s needs the range of services could vary from, e.g., daily telephone contacts, to services involving several caregivers such as home visits by primary care, rehabilitation by physiotherapists and others. The community care had staff on stand-by in case their services were needed, which generated costs. This led to many discussions about better planning in hospital discharges and on patient information needed to ensure patient safety.

*Proactive primary care* is a new service developed and tested in an intervention study aiming to evaluate whether the proactive approach can lead to a decrease in healthcare consumption and costs (Marcusson *et al*., 2019). Primary care centers implementing the proactive service are compared with a control group, who continued their work as usual. The intervention is still under evaluation. The service targets frail older people over 75 with comorbidity who are likely to be hospitalized. Primary care centers receive lists of patients, which are identified using a prediction model. A nurse contacts patients by telephone to make a first evaluation based on a standardized assessment tool. If a patient fulfills the criteria, a multidisciplinary care team is set up to create a CIP. One of the challenges is that the prediction model returns a high number of persons and contacting all of them to identify individuals with actual CIP needs requires considerable resources. The preliminary results show that a better prediction model needs to be developed if the proactive service is to be used in primary care.

### Work setting at home

Care provided at home requires more independent work skills from caregivers. Care professionals are more often left alone to perform their work and lack closeness to colleagues. A newly graduated physician claimed that home visits are very stressful, since there is no possibility to validate decisions with tests and consultations. At the hospital, physicians can usually observe patients for some time and order tests. At home, it may happen that the physician has never met the patient before. Accordingly, care professionals experience more responsibility and less medical control over the patient at home. Interviewees emphasized the importance of broad competence and clinical experience in nurses or assistant nurses who regularly visit an older person. In case medical intervention is needed, they become the physician's extended eyes. They can describe the older person and how the person’s status has changed. To act independently and have the ability to take decisions, was, therefore, considered important.

One returning theme in our study was the need to share *information across organizational settings.* Currently different patient record systems are used, which are often described as a major hindrance to providing safe and high-quality care at home. A single shared system is difficult to achieve due to the requirements of high security levels, secrecy and traceability when information is shared between different care settings. Interviewers described struggles in sharing information and problems related to prescriptions for medication and inappropriate supplies and materials. The solutions usually implied establishing standardized protocols for what and how information should be handled in a secure way. Efforts were also made to develop solutions at a systemic level. A digital platform called “The National Patient Overview” was implemented at national level. The platform (with the patient’s consent) automatically retrieves information from different record systems and authorized health and healthcare providers can share and access the information. Another example is “My Plans” which is an IT-supported system for joint work with CIP. Region Skåne developed a joint patient record system, which among others should enable process-oriented communication between all public and private health and social care providers and with the patients themselves.

Our empirical material also includes several examples of *technical aids* that are needed to support care at home. Examples range from medical technology that enable tests, investigations and treatments at home, such as mobile equipment for electrocardiograms, ultrasounds and spirometry; to technical solutions that enhance patient safety and quality of life, such as night cameras, home alarms, diapers with digital alert sensors and drug robots.

Technical aids can also be used for control and management of care services; for example, through a digital gauge forecasting the number of inpatient days before a patient returns home. Other examples are measurement and follow-up of mobile team's activities and results and a system to notify potential readmissions.

### Patients at home vs. in the hospital setting

Our findings suggest the hospital is considered the territory of healthcare professionals. There is dedicated equipment, access to laboratory tests and imaging and the possibility of medical consultations. The patient is usually a visitor who accepts the terms and conditions of hospital functioning and mostly follows what is undertaken without questioning decisions or medical practice. The focus is on diagnostics and treatment, so that the patient can be cured and discharged. Many medical procedures can be undertaken without the older patient’s understanding or consent. Below is a quote from a physician working in mobile team illustrating this issue:

Sometimes when you talk with older patients about hospital admissions, you ask: now you have been in the hospital three times this year, why it happened, why have you been in hospital? I do not know, I felt ill. Yes, but was it the heart or infection? I do not know; they did not tell me.

In contrast, home was considered to be the territory of older people. It was described in terms of a place filled with memories and feelings, free from disease and the image of oneself as a patient. Care staff are visitors and can’t impose requirements on the physical setting. The staff can only sensitively inform about perceived inconveniences and give advice about applying some aids. Additionally, daily routines as dressing, eating and cleaning become a natural part of the day and care services provided at home become a part of daily living rather than medical procedures. It was reported that care staff need to show respect for different lifestyles and values and see the whole person and not just a patient. A home care nurse described it as being ‘like a chameleon’:

I’m almost a different person depending on whose home I enter. I have to adapt to the patient in a different way compared to if I have a white coat and work at the hospital.

The care is to be provided on older person’s terms, placing integrity and dignity first and foremost. Interviewees emphasized the importance of asking and explaining planned medical interventions or adding new care services. This kind of dialog was described as more natural and easier in the home care setting. One of the physicians in the mobile team described that in many cases the older person does not want to take part in every offer for an examination or be admitted to the hospital because they lack the strength or do not want to continue with a treatment. It seems to be more difficult to see, accept and respect the older person's will during a hospital stay. “The healthcare staff may get a little speed blind” and be focused on putting out fires instead of assessing the holistic needs of the individual was another recurring theme.

In can be concluded that older people feel more confident and take more control over their own situation at home. Frequently there are also relatives who provide care and support in daily living. However, self-care is a regulated concept in Sweden and healthcare staff need to assess whether a certain care activity can be prescribed as self-care to a patient. The staff who have made this assessment are also responsible for informing, planning, documenting, following up and reconsidering self-care, as well as for educating the people who are supposed to help with self-care. One physician in our interviews expressed that caregivers are rather unwilling to give older people responsibility for self-care. The attitude seems to be “it will not work”, but the physician was often surprised at how well it ended up working. During the studies no examples of using the older person as a resource in their own care in a structured and systematic way emerged.

## Discussion

In this section the empirical findings are discussed with a focus on identifying development areas and connecting them with recent and previous research studies to provide state-of-the-art and future directions for home-centered-care. Despite the fact that the development initiatives studied in the paper represent best practices related to the successful implementation of policies towards person-centered and close care, they have a long journey to go before achieving the goals. We outline five propositions, which we consider playing a major role in supporting a comprehensive development strategy for designing and delivering home-centered care that is co-created with older people.

### Policy making for systemic integration

Our findings point to the importance of raising the administrative and legal barriers for home-centered care at national and regional levels. National policies create incentives for regions to develop solutions to achieve better integration between health and social care systems (Berglund *et al*., 2015). For example, the law of integrated discharge was one of the important reasons for forming an agreement between Region Skåne and related municipalities, which in turn enabled local solutions like discharge teams. At the same time national evaluations show that policies are not implemented to a sufficient extent. Although policymaking is considered important for driving improvement, it is unclear how best to ensure effective policy implementation in practice (Hudson *et al*., 2019). Policies formulated at the national level face the challenge of ensuring consistency in implementation at regional and organizational levels. The challenge is especially present in instances where the regional level, which is the case in Sweden, has an independent degree of political authority (Norris *et al*., 2014). Therefore, this paper calls for more attention to the *design of national policies that can enable home-centered care and research as to how policy implementation can be strengthened and supported.*

### Organizing for home-centered care

As hospital-centered care is structured, organized and managed to meet its aims and targets, appropriate organizational structure and managerial practices need to be developed to achieve high-quality, safe and effective home-centered care. Providing care at home is complex due to the diversity of services that need to be provided by different organizations with their own objectives, responsibilities and capabilities (Marino *et al*., 2018). Caregivers need to design and customize individual care offerings and deliver them as a coordinated and comprehensive set of services (Silander *et al*., 2018). The different solutions implemented by case organizations were not sufficient or were difficult to achieve and our findings indicate that we need to think in new directions to achieve integration of services provided to older patients. The research on modularity in healthcare offers a new perspective, as it allows for a configuration of services from standardized modules (Voss and Hsuan, 2009; Vähätalo and Kallio, 2015; Bartels *et al*., 2021). Modularity is an appealing approach, as it fits well with the reality of home-centered care. Caregivers provide different customized care services (modules), which need to be coordinated and co-created with older people (establishing interfaces) (De Blok *et al*., 2014). Our empirical data contains several examples of coordination and integration arrangements that can be described as interfaces from a modularity perspective. One example is the coordinators assigned to navigate and facilitate service provision. Another example is collaboration groups, which clarify individual caregivers' responsibilities and ensure that nothing falls between the cracks. Despite a few years of modularity research in healthcare there are few studies which were conducted on services provided at home. This leads to defining another important proposition for home-centered care: *focus on developing, implementing* *and* *evaluating mechanisms that enable care customization and connect and coordinate services between caregivers and with older people and relatives.*

### Expanding services that enable older people living at home

Today, healthcare needs to increasingly care for older persons with chronic conditions (Lillrank, 2018). In this paper we claim that in order to manage the challenges of increasing healthcare consumption and provide high-quality care of older people we have to make a shift from hospital-centered care to home-centered care. This doesn’t mean that all care is provided at home, but that care providers at primary, secondary and community levels think outside the box and develop care services that enhance older people’ ability to continue living and being cared for at home (WHO, 2016). Our empirical material contains examples of new services from all care settings that support this ability. Primary care units offer a proactive care service which is provided before hospitalization is required. A day care unit at a hospital offers a range of services that would usually imply hospitalization but are possible to carry out in outpatient settings. A mobile team offers in-home care services at primary and specialized care level. Finally, community care delivers a service that supports older patients at discharge. The new services need to be developed and offered by different care settings as individual modules that can be combined and customized to meet the older person’s individual needs (Bartels *et al*., 2021). The third proposition is therefore to *redefine the current services delivered by primary, secondary* *and* *community care and extend the scope of services that enable older people to live and co-create care at home as long as possible.*

### Developing human resources and infrastructure for home setting

Home-centered care differs in terms of required skills and knowledge by care employees and needs to be supported by appropriate human resources management practices and educational programs. Our findings show that care staff working at home need to be generalists rather than specialists. Broad medical or nursing knowledge and a few years' experience of working in institutional care is an advantage when providing care at home. Both international and Swedish studies have shown that home as a work setting is mentally and physically demanding (Strandell, 2020). At the same time our findings suggest that the relationship which can be developed between an older person and a caregiver can make the job more meaningful. As there is relationship between quality of care and quality of working life (Aiken *et al*., 2011) we have to focus on enhancing working conditions in home settings. An important factor identified in our study is the possibility of using medical technology that enables diagnostics and treatment at home and supports decision-making. A serious barrier is access to and sharing of information across organizational settings. The need to access a patient’s medical history is higher in chronic conditions compared to episodic care (WHO, 2016). This becomes an extra challenge when many care providers are involved and care is provided at home. Technical and digital aids are also important to ensure patient safety. Care provided at home is perceived as less safe compared with hospital care (Cao *et al*., 2021; Silverglow *et al*., 2021) and this problem can be, to some extent, addressed with technical and digital solutions. Therefore, the fourth proposition is to *understand the context of providing care at home and develop human resources and infrastructure that fit the home setting.*

### Realizing patient co-creation

The stories from the interviewees' professional experiences indicate that it is easier to see an older person as an individual and not only a patient at home compared to a hospital. The older person at home is more confident, better-informed and more likely to take control of their care. In the hospital-centered model the healthcare provider is often seen as the primary producer of value and the patient is seen as a passive recipient, or, at most, as co-producers of value (Elg *et al*., 2012). In contrast, in the home-centered model the older person should be seen as co-creator of value and caregivers can be seen as facilitators of the value creation process (McColl-Kennedy *et al*., 2017). This changed perspective is an important prerequisite for older person’s co-creation, which may uncover new or improved service opportunities. The study on CIP shows that many older people wish to be actively engaged in decisions regarding their care (Jobe *et al*., 2019). Another study by Engström *et al*. (2014) has shown that patients make different contributions to healthcare service development depending on the care setting (hospital vs. home), or if they are episodic or chronic patients. Our findings indicate that even though older people are perceived as more powerful and willing to participate in their own care at home, they are seldom seen as co-creators or the only creators of care services at home. Knowledge and information are no longer the sole propriety of healthcare professionals, as patients can educate themselves and use other actors offering services (e.g., monitoring apps). Therefore, the fifth and last proposition is *an increased focus on empowering and enabling older people and their relatives to co-create care services at home.*

## Practice implications

The findings of this paper are embedded in the Swedish context. The paper suggests home-centered care as a solution for caring for the increasing number of older people. The outlined propositions are not one size fits all solution, but a contribution to knowledge and practice on how to deliver care of older people across differentiated organizations within systems with different funding and regulatory structures. To achieve home-centered care, administrative and legal barriers hindering the integration of health and social system need to be lifted and appropriate organizational and managerial structures must be developed. Our recommendations include service modularity, which enables the customization and delivery of services in a multi-provider context; new services supporting home-centered care; the development of human resources management and technical aids for home settings; and an increased focus on patient–provider co-creation.

## Figures and Tables

**Table 1:** Care settings for older people in Sweden

Care level	Responsible provider	Type of services
*Community care*	290 Municipalities	Nursing homes
		In-home services; e.g., meals, cleaning
		Home nursing
		Home-based healthcare
*Primary care*	21 Regions	Health centers
*Secondary care*	21 Regions	Emergency, acute, and elective care provided in outpatient, day care, and inpatient settings

**Source(s):** Authors work

**Table 2:** Study design

Cases and levels studied		Data collection methods	Aspects studied	Key questions of analysis
*National level* Research project on “Policy development and deployment”		1 interview with national investigator at Ministry of Health and Social Affairs 11 documents studied from National Board of Health and Welfare, The Swedish Agency for Health and Care Services Analysis and The Swedish Association of Local Authorities and Regions	Policy changes related to care of older people	Which of the policy changes contribute to home-centered care?
*Regional level* Research project on “Policy development and deployment”	*Region Skåne*	3 interviews with managers responsible for elderly care 17 documents studied	Actions related to policy deployment	How to achieve policy deployment at the local level? Which actions were important for home-centered care?
	*Region* *Östergötland*	2 interviews with managers responsible for elderly care 18 documents studied		
*Local level* Research project on “Innovations and new thinking in providing care of older people”	*Case:* *“Health city*”	5 interviews with managers responsible for hospital, primary and community care 7 documents studied	Organizational integration of care settings, coordination, discharge teams, and mobile teams	What was done to integrate and coordinate services? What were facilitators and inhibitors for home-centered care?
	*Case:* *“Future care”*	22 interviews with professionals from day care unit 10 interviews with professionals from primary care, home care and mobile teams 25 meeting observations 4 documents studied	Operative collaboration group, Day care unit Mobile teams Discharge teams, and in-home services	How do care settings and the patient's role differ at home compared to hospital? What were facilitators and inhibitors for home-centered care?
	*Case:* *“Proactive primary care”*	11 interviews with mangers of primary care centers 2 documents studied	Activities related to the new proactive service targeting frail older persons over 75 (Prediction list, CIP, contacts with patients)	What were facilitators and inhibitors of providing the service?

**Source(s):** Authors work
